# A Putative Transcription Factor MYT2 Regulates Perithecium Size in the Ascomycete *Gibberella zeae*

**DOI:** 10.1371/journal.pone.0037859

**Published:** 2012-05-23

**Authors:** Yang Lin, Hokyoung Son, Kyunghun Min, Jungkwan Lee, Gyung Ja Choi, Jin-Cheol Kim, Yin-Won Lee

**Affiliations:** 1 Department of Agricultural Biotechnology and the Center for Fungal Pathogenesis, Seoul National University, Seoul, Republic of Korea; 2 Department of Applied Biology, Dong-A University, Busan, Republic of Korea; 3 Eco-friendly New Materials Research Group, Research Center for Biobased Chemistry, Division of Convergence Chemistry, Korea Research Institute of Chemical Technology, Daejeon, Republic of Korea; University of Wisconsin – Madison, United States of America

## Abstract

The homothallic ascomycete fungus *Gibberella zeae* is a plant pathogen that is found worldwide, causing Fusarium head blight (FHB) in cereal crops and ear rot of maize. Ascospores formed in fruiting bodies (i.e., perithecia) are hypothesized to be the primary inocula for FHB disease. Perithecium development is a complex cellular differentiation process controlled by many developmentally regulated genes. In this study, we selected a previously reported putative transcription factor containing the Myb DNA-binding domain MYT2 for an in-depth study on sexual development. The deletion of *MYT2* resulted in a larger perithecium, while its overexpression resulted in a smaller perithecium when compared to the wild-type strain. These data suggest that MYT2 regulates perithecium size differentiation. *MYT2* overexpression affected pleiotropic phenotypes including vegetative growth, conidia production, virulence, and mycotoxin production. Nuclear localization of the MYT2 protein supports its role as a transcriptional regulator. Transcriptional analyses of trichothecene synthetic genes suggest that MYT2 additionally functions as a suppressor for trichothecene production. This is the first study characterizing a transcription factor required for perithecium size differentiation in *G. zeae*, and it provides a novel angle for understanding sexual development in filamentous fungi.

## Introduction

The homothallic ascomycete fungus *Gibberella zeae* (anamorph: *Fusarium graminearum*) is a worldwide plant pathogen that causes Fusarium head blight (FHB) in cereal crops and ear rot of maize [1]. This fungal infection leads to severe yield losses and the accumulation of mycotoxins, such as trichothecenes and zearalenone, which are harmful to humans and livestock [2]. *G. zeae* produces ascospores (sexual spores) and conidia (asexual spores), and both are considered as disease inocula. However, the ascospores formed in fruiting bodies (i.e., perithecia) are proposed to be the primary inocula for FHB. After overwintering as perithecia or perithecia-associated hyphae formed on plant debris, the ascospores are forcibly discharge from mature perithecia during flowering season and are then considered primary inocula [3], [4], [5], [6].

Perithecia are complex multicellular structures that protect sexual spores and ensure their proper discharge [7]. Perithecial morphogenesis can be conveniently divided into three morphologically distinct developmental stages: ascogonial, protoperithecial, and perithecial [8]. Homothallic fungi usually generate female reproductive structures called ascogonia, which further develop into spherical protoperithecia. The tips of ascogenous hyphae contain two nuclei that pair to form the dikaryotic state. This dikaryotic mycelial phase is followed by karyogamy of two haploid nuclei, resulting in a diploid nucleus. The formation of a diploid nucleus is a prerequisite to meiotic division. After meiosis, the four haploid nuclei undergo a postmeiotic mitosis. As a result, every ascus contains eight nuclei, and each nucleus is a starting point for ascospore formation [9], [10]. Although homothallic fungi may not require the mating process, the nuclei are required to pair and form a dikaryon within the ascogenous hyphae [11]. In *G. zeae*, a previous in-depth microscopic observation of sexual development was unable to identify binucleate hyphae during the initiation of the sexual stage [4]. The binucleate condition was eventually established in the ascogenous hyphae and first observed in the crosiers [4].

The development of perithecia in filamentous ascomycetes is a complex cellular differentiation process that is under polygenic control [12], [13]. The cytology and genetics of ascus development, meiotic silencing by unpaired DNA, and ascospore formation have been studied in considerable detail in *Neurospora crassa*, *Sordaria macrospora*, and *G. zeae* [10], [11], [12], [14], [15], [16]. Some genes identified in these species are now known to be involved in the formation of the perithecia that arise during sexual propagation. Most of these genes take part in signal transduction cascades, transcriptional or posttranscriptional regulation, and primary or secondary metabolism [17]. In *S. macrospora*, several genes governing the transition from the spherical protoperithecial stage to the flask-shaped perithecial stage have been studied at the molecular level. However, few molecular and biochemical details are known about factors governing this differentiation process [9].

In *G. zeae*, several genes and pathways are considered to play important roles in perithecial development, including mating type genes [18], [19] and G-protein and MAP-kinase signaling pathways [20], [21], [22], [23]. The release of a genome sequence assembly for *G. zeae* has allowed the use genome-wide approaches for identifying more genetic elements involved in the sexual reproduction process [24], [25], [26], [27], [28], [29], and forward and reverse genetics-based studies have found many sexual development-related genes [30], [31], [32], [33], [34], [35], [36], [37], [38], [39], [40], [41]. With the exception of *ROA*, null mutants of these genes consistently show defects in the pleiotropic phenotype such as mycelia growth, conidiation, toxin production, virulence, and sexual development.

As a ubiquitous family, proteins containing the Myb DNA-binding domain play diverse roles in eukaryotes, and this domain is typically found in eukaryotic transcription factors. After the identification of the first Myb domain-containing protein, v-Myb of the avian myeloblastosis virus [42], researchers subsequently found several Myb domain-containing proteins, such as c-*myb*, A-*myb*, B-*myb* and their homologs in vertebrates, insects, fungi, and slime molds{Klempnauer, 1982 #8300} [43], [44]. In animals, Myb family members are considered to be oncogenes involved in colon and breast cancer and some human leukemias [45] and to play various roles in the control of cell proliferation, apoptosis, and differentiation [46]. In plants, MYB proteins are a superfamily of transcription factors that regulate networks controlling primary and secondary metabolism, cell fate and identity, developmental processes, and responses to biotic and abiotic stresses [47]. In fungi, even though only a few Myb-related proteins have been reported, the functions of these proteins are various and include G2/M progression and pre-mRNA splicing (cdc5p), termination of rRNA transcription and G1 arrest in response to nitrogen starvation (Reb1), and activation of GCN4-indepentdent *HIS4* transcription (BAS1) [48], [49], [50]. The Myb-related gene *FlbD* was reported in filamentous fungi and is known to control conidiophore development in *Aspergillus nidulans* [51], [52]. Recently, we found a MYT1 transcription factor containing a Myb domain that is involved in female fertility in *G. zeae* [41].

Previously, we performed genome-wide functional analyses of whole transcription factor genes in *G. zeae* [29]. In this study, we selected one gene that previously demonstrated a defect in perithecial development and further characterized its function in *G. zeae* using a variety of techniques, including gene deletion and overexpression.

## Methods

### Fungal strains and media

All of the strains used in this study are listed in Table 1. Conidia and mycelia of the wild-type strain Z-3639 [53] and mutants derived from this wild-type strain were stored in 20% glycerol at −70°C. A transgenic strain mat1r carrying both a *MAT1–1* deletion and histone H1 tagged with red fluorescence protein (RFP), was used for the co-localization study, as previously described [36]. A minimal medium containing 5 mM agmatine (MMA) was used to evaluate trichothecene production [54]. Yeast malt agar (YMA) was used to induce conidia production as previously described [55]. All of the other media used in this study were prepared as per the *Fusarium* laboratory manual [1].

**Table 1:** *G. zeae* strains used in this study.

Strain	Genotype	Source or reference
Z-3639	Wild-type	[53]
myt2	Δ*myt2::gen*	[29]
MYT2com	Δ*myt2::MYT2-GFP-hyg*	This study
MYT2OE	*MYT2::gen-_ PEF1α_ -MYT2*	This study
mat1r	Δ*mat1-1::gen; hH1::hH1-RFP-gen*	[36]
MYT2comr	Δ*myt2::MYT2-GFP-hyg; hH1::hH1-RFP-gen*	This study

### Nucleic acid manipulations, primers, and sequencing

Fungal genomic DNA was prepared as previously described [1]. The mycelia or perithecia in different stages were harvested, and total RNA was isolated using the Easy-Spin Total RNA Extraction Kit (Intron Biotech, Seongnam, Korea). Restriction endonuclease digestion, agarose gel electrophoresis, and DNA gel blot hybridization with ^32^P-labeled probes were performed following standard protocols [56]. PCR primers were synthesized at an oligonucleotide synthesis facility (Bionics, Seoul, Korea) (Table S1) and stored at −20°C at a concentration of 100 μM. General PCR reactions were processed following the manufacturer's instructions (Takara Bio Inc., Otsu, Japan). DNA sequencing was performed by Macrogen Korea (Seoul, Korea), and sequences were compared against the *Fusarium* Comparative Database at the Broad Institute (http://www.broadinstitute.org/annotation/genome/fusarium_graminearum).

### Rapid amplification of cDNA ends (RACE)-PCR

We determined the *MYT2* open reading frame (ORF) using rapid amplification of cDNA ends (RACE)-PCR. The cDNA library used as template was constructed in a previous study [36]. Three fragments located around the *MYT2* ORF were amplified with MYT2-seq1/MYT2-seq2, pPRN3-N-For/MYT2-seq2, and pPRN3-N-Rev/MYT2-seq1 primers and then directly sequenced.

### Genetic manipulations and fungal transformations

We applied a slightly modified double-joint (DJ) PCR strategy [57] to construct fusion PCR products for complementation and overexpression. To complement the *MYT2* deletion mutant (Δ*myt2*), the *MYT2* ORF was fused with green fluorescent protein (GFP) by DJ PCR as previously described [36]. In brief, the *MYT2* ORF with its own promoter was fused with *GFP*-*hyg*, carrying the *GFP* gene and the hygromycin resistance gene cassette (*hyg*) amplified from pIGPAPA [58], and the 3′ flanking region of the *MYT2* gene. Using this PCR product as a template, a final fusion construct was amplified with the nested primer pair MYT2–5N/MYT2–3N. Finally, we transformed the fusion construct into the myt2 mutant strain.

To overexpress *MYT2*, we generated a fusion construct containing the 5′ flanking regions of *MYT2*, the *MYT2* ORF, and the *gen-P_EF1α_*-carrying elongation factor 1α promoter (*P_EF1α_*) from *Fusarium verticillioides* as previously described [37]. The *gen-P_EF1α_* sequence was amplified from pSKGEN [37] with primers Neo-for new and eGFP-P1. The 5′ flanking regions of *MYT2* and the *MYT2* ORF were amplified by primer pairs MYT2–5F/MYT2–5R OE and MYT2–3F OE/MYT2–3R OE, respectively. Three amplicons were then fused by a secondary round of DJ PCR. Using this fusion fragment as a template, a final PCR product was amplified with the nested primers MYT1–5N and MYT1–3N OE. Finally, this fusion construct was transformed into the wild-type strain.

### Quantitative real-time (qRT)-PCR

To obtain the *MYT2* expression profile in different *G. zeae* strains, we extracted the total RNA of each strain from vegetative cultures at 5 d after inoculation and sexual cultures at 3, 5, and 7 d after sexual induction, respectively. We then synthesized the first strand of cDNA from the total RNA with SuperScriptIII reverse transcriptase (Invitrogen, Carlsbad, CA, USA). qRT-PCR was performed using SYBR Green Super mix (Bio-Rad, Hercules, CA, USA) and a 7500 real-time PCR system (Applied Biosystems, Foster City, CA, USA) with the MYT2-realtime-F/MYT2- realtime-R primers (Table S1). For normalization, the cyclophilin gene (*CyP1*; locus ID: FGSG_07439.3) was used as an endogenous control [59]. The PCR was repeated three times with two replicates per run. The relative transcript level of *MYT2* in each strain was calculated as previously described [60]. Briefly, gene expression was calibrated using the formula 2^-ΔΔ*C*^ *_T_* method. The threshold cycle (*C_T_*) value of *CyP1* was subtracted from that of *MYT2* to obtain a Δ*C_T_* value. The Δ*C_T_* value of *MYT2* expression in the wild-type strain at the 5-d vegetative stage was subtracted from the Δ*C_T_* value of each sample to obtain a ΔΔ*C_T_* value. The MYT2 expression level relative to the calibrator was obtained as 2^-ΔΔ*C*^ *_T_*. Significant differences (*p*<0.05) of 2^-ΔΔ*C*^ *_T_* were examined statistically among the mean values of the samples based on Tukey's test.

**Figure 1:**
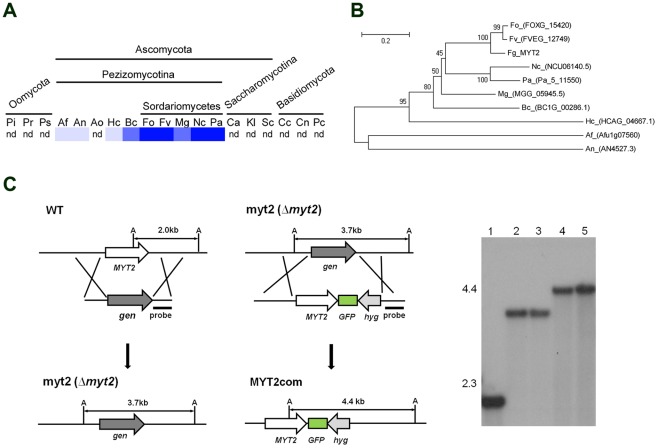
Distribution of MYT2 homologs in fungi and genetic complementation. (A) Distribution of MYT2 in representative fungal species. The distribution image was constructed using the BLASTMatrix tool that is available on the Comparative Fungal Genomics Platform (http://cfgp.riceblast.snu.ac.kr/) [72]. (B) Phylogenetic tree of MYT2 homologs in several fungal species. The alignment was performed with ClustalW, and the MEGA program, version 4.0, was used to perform a 1,000-bootstrap phylogenetic analysis using the neighbor-joining method. Pi, *Phytophthora infestans*; Pr, *P. ramorum*; Ps, *P. sojae*; Af, *Aspergillus fumigatus*; An, *A. nidulans*; Ao, *A. oryzae*; Hc, *Histoplasma capsulatum*; Bc, *Botrytis cinerea*; Fo, *Fusarium oxysporum*; Fv, *F. verticillioides*; Mg, *Magnaporthe grisea*; Nc, *Neurospora crassa*; Pa, *Podospora anserine*; Ca, *Candida albicans*; Kl, *Kluyveromyces lactis*; Sc, *Saccharomyces cerevisiae*; Cc, *Coprinus cinereus*; Cn, *Cryptococcus neoformans*; Pc, *Phanerochaete chrysosporium*; nd, not detected. (C) Targeted deletion and complementation of *MYT2*. WT, *G. zeae* wild-type strain Z-3639; myt2, *MYT2* deletion mutant; MYT2com, myt2*-*derived strain complemented with *MYT2-GFP*; A, *Ava*I; *gen*, geneticin resistance gene cassette; *hyg*, hygromycin B resistance gene cassette. Lane 1, Z-3639; lanes 2 and 3, *MYT2* mutants; lanes 4 and 5, MYT2com mutants. The sizes of the DNA standards (kb) are indicated to the left of the blot.

To confirm whether *MYT2* regulates the toxin synthesis-related genes, *Tri5* and *Tri6*, we incubated the conidia of wild-type, myt2, and MYT2OE strains in MMA media for 5 d and isolated total RNA from these cultures. We synthesized first-strand cDNA and performed qRT-PCR for the *Tri5* and *Tri6* genes as described above.

### Sexual crosses

Each strain was incubated on carrot agar [1] at 25°C for 5 d. Mycelia grown on carrot agar were mock fertilized with 700 μl of 2.5% Tween 60 solution to induce fertilization and were then incubated under a near-UV light (wavelength: 365 nm, HKiv Import & Export Co., Ltd., Xiamen, China) at 25°C. Seven days after induction, the perithecia from each strain were dissected in a drop of 15% glycerol. The cell size of perithecia and asci rosettes within the perithecia were observed under a DE/Axio Image A1 microscope (Carl Zeiss, Oberkochen, Germany). Nine days after sexual induction, we collected ascospores discharged from the perithecia of each strain and measured the number of septa and the length and width of the ascospores using the same microscope.

We also counted the number of ascospores per perithecia for each strain as previously described [34]. Each strain was inoculated on carrot agar and was mock fertilized. The circular agar block (14.5 mm in diameter) of each strain was downwards fixed on the lid of a 24-well plastic plate (SPL Lifesciences, Pocheon, Korea) 7 d after sexual induction, but before the ascospores were discharged from the perithecia, and incubated at 25°C for another 7 d. Ascospores within the perithecia can be completely discharged onto the plastic plate. All discharged ascospores were collected from the 24-well plastic plate through 14 d after sexual induction with 500 μl of sterile distilled water and were counted with a haemacytometer (Superior Co., Germany). After counting the number of perithecium on each block, the total ascospore number per perithecium was obtained. The experiments were performed three times with three replicates.

### Conidia production, morphology, and germination

After a 72-h incubation in 50 ml of complete media (CM) at 25°C on a rotary shaker (150 rpm), the mycelia of each strain were harvested and washed twice with sterile distilled water. To induce conidiation, the mycelia were spread onto YMA plates and incubated at 25°C under near-UV light. After 48 h, the conidia that had formed on the YMA were collected with sterile distilled water, filtered through cheese cloth, washed with sterile distilled water, and centrifuged (5000 rpm, 25°C, 5 min). A 1-ml conidia suspension (1×10^5^ conidia ml^−1^) of each strain was inoculated into 50 ml of CMC and then incubated at 25°C on a rotary shaker (150 rpm). The number of conidia produced after a 3-d incubation in CMC media was counted to measure conidia production by each strain.

To observe conidial morphology, the conidia produced by each strain on YMA were harvested and stained with Calcofluor white stock solution (10 mg ml^–1^; Sigma, 18909). Microscopic observation was performed with a DE/Axio Imager A1 microscope (Carl Zeiss) using the filter set 49 (excitation 356; emission 445/50), the number of septa was counted, and the length and width of the conidia were measured.

The conidia germination rate was counted as previously described [60]. A 1-ml conidia suspension (1×10^7^ conidia ml^−1^) harvested from YMA medium was inoculated into 20 ml of CM or minimal medium (MM). The number of germinated conidia per 200 conidia was counted after incubation at 0, 2, 4, 6, and 8 h. The experiment was performed twice with three replicates for each point.

### Virulence test and trichothecene analysis

To test the virulence of each strain on wheat head, the point inoculation method was performed as previously described [33]. The conidia of each strain were harvested from CMC and adjusted to 10^5^ conidia ml^−1^. Thereafter, 10 μl of each conidial suspension was injected into a center spikelet of the wheat head (cultivar Eunpamil) at mid-anthesis. The wheat plants were then incubated in a humidity chamber for 3 d and transferred to a greenhouse. The number of spikelets showing disease symptoms was counted 14 d after inoculation as previously described [36]. The experiment was performed with five replicate inoculations per strain, and two independent mutant strains were used for the experiment.

The trichothecene analysis was performed as previously described [36]. Briefly, cultures grown in MMA were filtered with cheese cloth and extracted with ethyl acetate. We then concentrated the extracts to dryness. Derivatization of each dry extract was performed with Sylon BZT (BSA + TMCS + TMSI at a 3∶2∶3 ratio, respectively; Supelco, Bellefonte, PA, USA), and the derivatized products were analyzed using a Shimadzu QP-5000 gas chromatograph mass spectrometer (GC-MS, Shimadzu, Kyoto, Japan) with a selected ion-monitoring mode as previously described [61]. We quantified the total trichothecene concentration based on the biomasses produced by each strain in MMA. The experiment was repeated three times.

### MYT2-GFP localization

We constructed a strain carrying both *MYT2*-*GFP* and *hH1-RFP-gen* to observe co-localization of MYT2 with nuclei using an outcross between the mat1r strain [36] and the MYT2com strain. After fertilizing the mat1r strain with the MYT2com strain, we performed a single-spore isolation. Ascospores carrying both *MYT2*-*GFP* and *hH1-RFP-gen* were selected using antibiotic resistance and confirmed by PCR assays. We observed localization of the fluorescence signal in cultures grown from CM, MM, carrot agar, and CMC. Microscopic observation was performed with a DE/Axio Imager A1 microscope (Carl Zeiss) using the filter set 38HE (excitation 470/40; emission 525/50) for GFP and the filter set 15 (excitation 546/12; emission 590) for RFP.

## Results

### MYT2 identification

We performed a RACE-PCR and determined that the transcription and splicing of FGSG_07546.3 *in vivo* were the same as the deduced ORF in the database. We designated FGSG_07546.3 as Myb DNA-binding domain-containing transcription factor 2 (*MYT2*), which encodes a 323-amino acid polypeptide containing the Myb DNA-binding domain. With the exception of the Myb DNA-binding domain, no other known motif exists in MYT2. MYT2 has no orthologs in the species of the phyla Oomycota and Basidiomycota, but it is conserved in species of the subphylum Pezizomycotina of the Ascomycota, particularly in Sordariomycetes (Figure 1A and B). However, none of the *MYT2* orthologs have been functionally characterized in other fungi.

### Complementation and overexpression

A construct carrying *MYT2-GFP* was introduced into the previously generated *MYT2* deletion mutant [29] for genetic complementation with GFP tagging as previously described [36] to generate the MYT2com strain. Deletion and complementation were confirmed by Southern hybridizations (Figure 1C).

**Figure 2:**
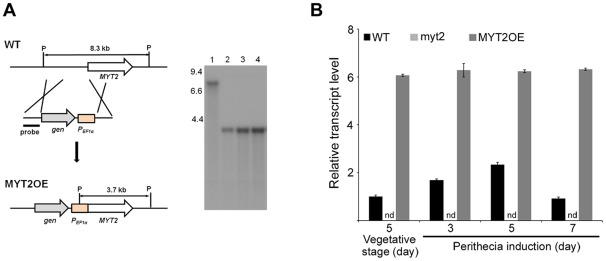
*MYT2* overexpression. (A) The *MYT2* promoter region was replaced with the *EF1α* promoter. The left and right panels show the strategy of MYT2OE strain construction and Southern hybridization, respectively. In the blot, lane 1 and lanes 2–4 represent the wild-type strain and the *MYT2*-overexpressed mutants, respectively. (B) Expression of *MYT2* in the wild-type, *MYT2* deletion, and *MYT2* overexpression strains. *MYT2* transcript accumulation was analyzed by quantitative real time-PCR (qRT-PCR) during the vegetative and sexual induction stages. WT, wild-type strain Z-3639; MYT2OE, transgenic strain where the *MYT2* promoter region was replaced with the *EF1α* promoter; P, *Pst*I. The sizes of DNA standards (kb) are indicated to the left of the blot.

We also generated a *MYT2*-overexpression strain (MYT2OE) in which *MYT2* expression was controlled by inserting the *EF1α* promoter (Figure 2A). Southern hybridizations were performed to confirm all genetic manipulations (Figure 2A). The *MYT2* transcript level in each strain was confirmed by qRT-PCR. In the wild-type strain, the *MYT2* expression level was significantly up-regulated at 3 d after sexual induction, increased until 5 d, and decreased again at 7 d. During both the vegetative growth and sexual development stages, the *MYT2* expression level in the myt2 strain was undetectable, but it was constitutively up-regulated in the MYT2OE strain by approximately fivefold compared to the wild-type strain (Figure 2B).

**Figure 3:**
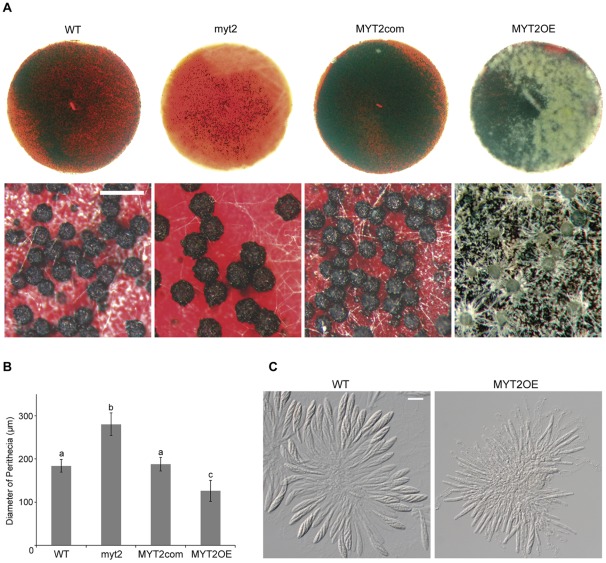
Self-fertility and asci rosettes of the *G. zeae* strains. (A) Perithecia of the *G. zeae* strains. Five-day old carrot agar culture was mock-fertilized to induce sexual reproduction and incubated for an additional 7 d. The upper and lower panels show the photographs of perithecia formed on a whole carrot agar plate and the photographs taken with a dissecting microscope, respectively. Scale bar  = 200 µm. (B) Diameter of the perithecia of the *G. zeae* strains. The diameters of 300 perithecia were measured for each strain using a dissecting microscope. Values with different letters are significantly different (*p*<0.05) based on Tukey's test. (C) Asci rosettes of wild-type and *MYT2* overexpression strains. Perithecia were dissected seven days after sexual induction. Scale bar  = 20 µm. WT, *G. zeae* wild-type strain Z-3639; myt2, *MYT2* deletion mutant; MYT2com, myt2*-*derived strain complemented with *MYT2*; MYT2OE, transgenic strain that has the *EF1α* promoter in place of the *MYT2* promoter region.

**Table 2:** Production and morphology of conidia and ascospores.

Strain	Conidiation (number/ml)^a^	Conidia^b^			Ascospores^c^		
		Length	Width	Septa	Length	Width	Septa
WT	1.7×10^6^A	48A	4.8A	4.5A	23A	5.0A	2.4A
myt2	1.7×10^6^A	49A	4.9A	4.5A	22A	5.3B	2.5A
MYT2com	1.8×10^6^A	48A	4.9A	4.5A	22A	4.9A	2.4A
MYT2OE	0.7×10^5^B	39B	4.7B	3.9B	20B	4.9A	1.4B

a Conidiation was measured by counting the number of conidia produced after a 3-d incubation in CMC.

b Macroconidia were produced on YMA. A total of 100 macroconidia were observed for each examination.

c Ascospores were collected from culture plate lids 10 d after sexual induction. A total of 300 ascospores were observed for each examination.

d All experiments were repeated three times with three replicates each. Values within a column with different letters are significantly different (*p*<0.01) based on Tukey's test.

### Sexual development

The previously described mutant phenotypes of *MYT2* deletion mutants include defects in perithecia formation with normal ascospore formation [29]. Further in-depth examinations were performed in this study. The selfing of the *MYT2* deletion strain (myt2) resulted in larger perithecia but approximately 7% of the number of perithecia produced by the wild-type and complemented strains (Figure 3A). The average perithecium diameter produced by myt2 was approximately 280 μm, which was nearly 1.5-fold larger than those produced by the wild-type and complemented strains (*p*<0.05). By contrast, the average perithecium diameter produced by the MYT2OE strain was approximately 0.7-fold smaller than those produced by the wild-type strain (*p*<0.05) (Figure 3B).

**Figure 4:**
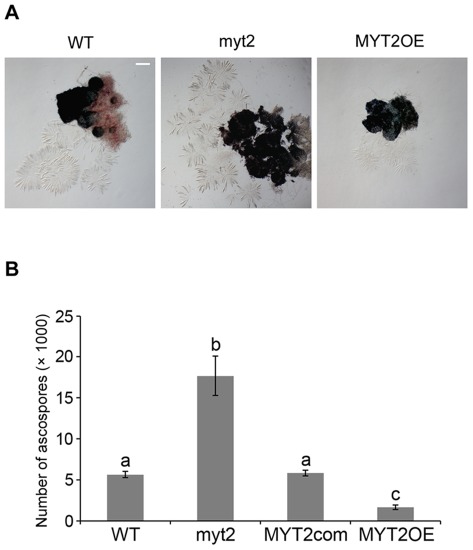
Asci rosettes and ascospores per perithecium of the *G. zeae* strains. Each strain was inoculated on carrot agar and was mock fertilized. (A) The perithecia from each strain were softly squeezed with cover slides to exude whole asci rosettes. The picture of each strain is representative of more than 20 repetitions. (B) All discharged ascospores were collected from the culture plate through 14 days after sexual induction. The number of ascospores per perithecia was obtained by dividing the number of perithecia by the number of discharged ascospores. Values with different letters are significantly different (*p*<0.05) based on Tukey's test. WT, *G. zeae* wild-type strain Z-3639; myt2, *MYT2* deletion mutant; MYT2com, myt2*-*derived strain complemented with *MYT2*; MYT2OE, transgenic strain that has the *EF1α* promoter in place of the *MYT2* promoter region.

The cell size of perithecia formed by wild-type and mutant strains was not significantly different (data not shown). Both the myt2 and MYT2com strains formed mature ascospores inside the asci that were similar to the wild-type strain 7 d after sexual induction. Although the MYT2OE mutant showed delayed ascospore maturation, it normally matured and discharged after 3–5 d later (Figure 3C). Ascospores produced by the myt2 mutant were wider than the wild-type strain, and the length and number of septa of the ascospores produced by the MYT2OE mutant were reduced compared to the wild-type strain (Table 2). Thus, myt2 mutants produce larger ascospores, while the MYT2OE mutant produces smaller ascospores.

**Table 3:** Radial growth and germination rate.

Strain	Radial growth (mm)^a^		Germination (%)^b^	
	CM^c^	MM	CM	MM
WT	74A^d^	80A	86A	48A
myt2	79B	84B	90A	54A
MYT2com	73A	82A	85A	45A
MYT2OE	61C	58C	86A	42A

a Radial growth was measured after a 5-d incubation.

b The germination percentage was measured after a 6-h incubation.

c CM, complete medium; MM, minimal medium.

d All experiments were repeated three times with three replicates each. Values within a column with different letters are significantly different (*p*<0.05) based on Tukey's test.

The myt2 and MYT2OE strains contained more and less asci rosettes compared to the wild-type strain, respectively (Figure 4A). The average ascospore number per perithecium in the myt2 mutant was approximately 3-fold greater than the wild-type strain (*p*<0.05), while the number for the MYT2OE mutant was approximately 0.3-fold less (*p*<0.05) (Figure 4B). This result was similar to the volume ratio of the perithecia from each strain (1∶3.5∶0.4 for the wild-type strain, the deletion mutant, and the overexpression mutant, respectively), which was calculated based on the diameter assuming the perithecium was a complete globular-shaped structure.

**Figure 5:**
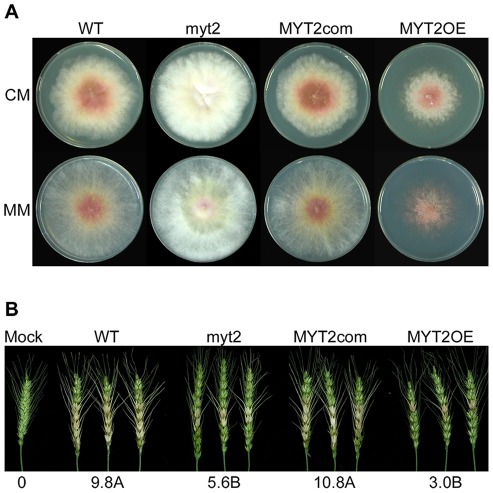
Mycelia growth and wheat head virulence of the *MYT1* mutants. (A) Mycelial growth on complete media (CM) and minimal media (MM) 5 d after inoculation. (B) A center spikelet of each wheat head was injected with 10 µl of conidia suspension. Values with different letters are significantly different (*p*<0.05) based on Tukey's test. Mock, negative control mock inoculated with 0.01 % Tween 20; WT, *G. zeae* wild-type strain Z-3639; myt2, *MYT2* deletion mutant; MYT2com, myt2*-*derived strain complemented with *MYT2*; MYT2OE, transgenic strain that has the *EF1α* promoter in place of the *MYT2* promoter region.

**Figure 6:**
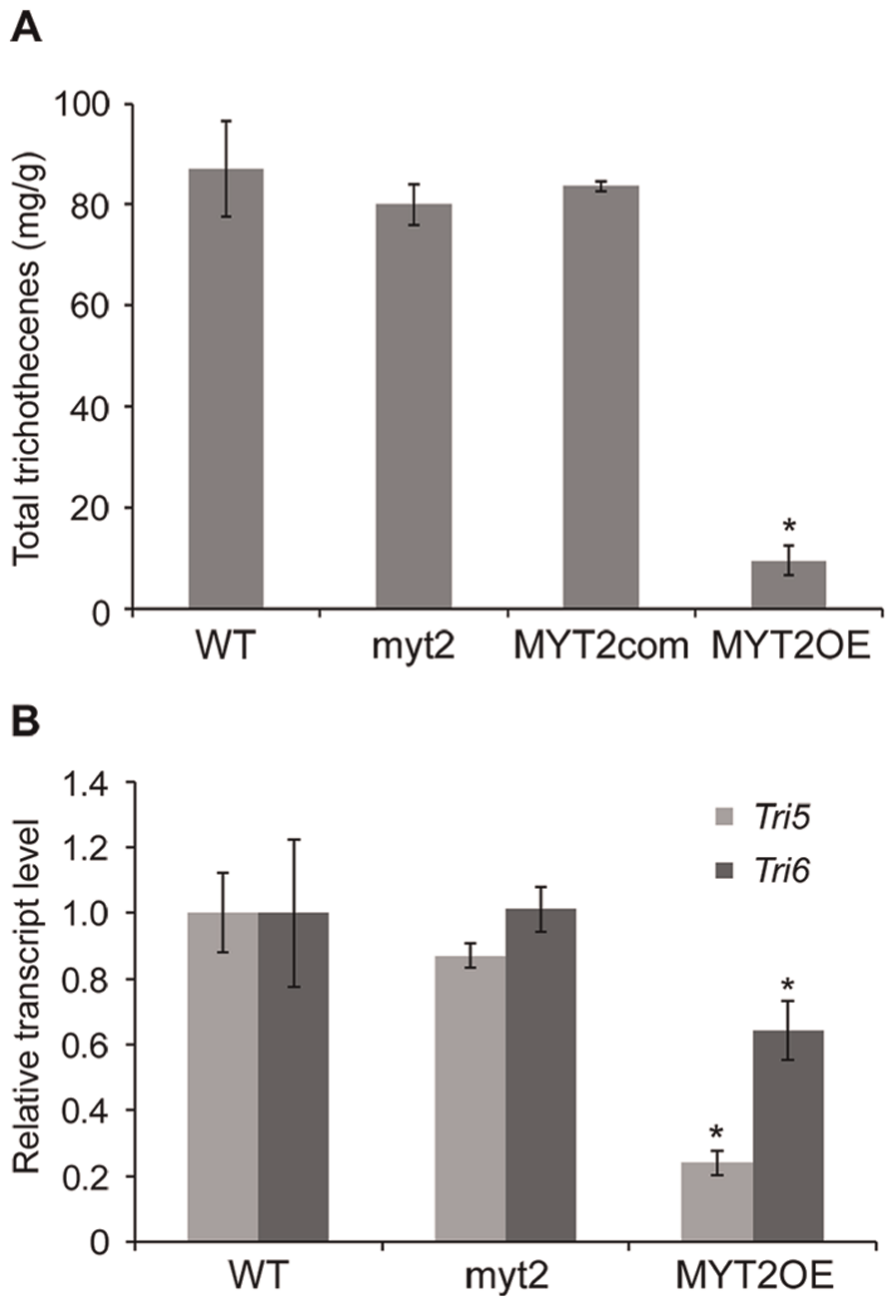
Total trichothecene (deoxynivalenol and 15-acetyldeoxynivalenol) production and transcriptional analyses of trichothecene synthetic genes. (A) Each strain was grown in minimal medium supplemented with 5 mM agmatine for 7 d. Trichothecenes were analyzed by GC-MS and quantified based on the biomass produced by each strain. Asterisks indicate data that significantly differed (*p*<0.05) based on Tukey's test (B) Expression of *Tri5* and *Tri6* in the wild-type, *MYT2* deletion, and *MYT2* overexpression strains. Gene transcription was analyzed by quantitative real time-PCR (qRT-PCR) 4 d after inoculation in MMA. WT, *G. zeae* wild-type strain Z-3639; Δ*myt2*, *MYT2* deletion mutant; MYT2com, Δ*myt2*-derived strain complemented with *MYT2*; MYT2OE, transgenic strain that has the *EF1α* promoter inserted in place of the *MYT2* promoter region.

**Figure 7:**
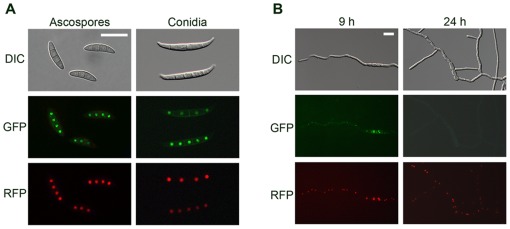
Cellular localization of MYT2. MYT2 was fused with green fluorescent protein (GFP), and histone H1 was fused with red fluorescent protein (RFP). Co-localization of MYT2-GFP and hH1-RFP in spores (A) and germinated conidia 9 and 24 h after inoculation in complete medium (B). DIC, differential interference contrast Scale bar  = 20 µm.

### Conidia production and germination

After a 3-d incubation in CMC media, there was no significant difference in conidial production among the wild-type, myt2, and MYT2com strains. However, the MYT2OE strain only produced 4% of the number of conidia produced by the wild-type strain (*p*<0.05) (Table 2). Similar to the ascospore morphology, the length, width, and number of septa of the MYT2OE mutant conidia were significantly reduced compared to the other strains (*p*<0.05) (Table 2). No significant difference was detected among the wild-type, myt2, MYT2com, and MYT2OE strains for conidial germination at 6 h after incubation in CM or MM (Table 3).

### Vegetative growth, virulence, and trichothecene production

The *MYT2* deletion mutant grew faster and produced more aerial mycelia than the wild-type and complemented strains and accumulated a low level of red pigment in both CM and MM. By contrast, the MYT2OE strain had a severe defect in vegetative growth (Figure 5A).

At 14 days after wheat head inoculation, the wild-type and MYT2com strains caused typical head blight symptoms, while both the myt2 and MYT2OE strains showed reduced virulence compared to the wild-type and MYT2com strains (*p*<0.05). The symptoms merely spread to nearby spikelets on the same wheat heads for the myt2 and MYT2OE strains (Figure 5B).

The level of trichothecene synthesized by both the myt2 and MYT2com strains was not significantly different than that of the wild-type strain. However, the MYT2OE mutant produced a significantly reduced level of trichothecene (Figure 6A). Transcription of the trichothecene synthetic genes *Tri5* and *Tri6* was also significantly reduced in the MYT2OE strain (Figure 6B).

### MYT2-GFP localization

To examine MYT2 localization, the *MYT2-GFP* fusion construct under the control of its native promoter was transformed into the *MYT2* deletion mutant. We selected six MYT2com strains carrying a single *MYT2-GFP* copy and found a GFP signal in the nuclei of all of the examined strains. To confirm nuclear localization of MYT2-GFP, MYT2comr (Δ*myt2::MYT2-GFP-hyg; hH1-RFP-gen*) was generated by an outcross between mat1r [36] and MYT2com. MYT2-GFP in the MYT2comr strain co-localized with hH1-RFP and was highly fluorescent in conidia and ascospores (Figure 7A). However, the GFP signals became blurred after germination and were undetectable 24 h later (Figure 7B).

## Discussion

The Myb DNA-binding domain is typically found in eukaryotic transcription factors. Previous reports demonstrated that Myb gene family members play diverse roles as transcriptional regulators in multiple cellular processes in animals and plants, including cell proliferation, apoptosis, differentiation, metabolic pathways, cell fate and identity, and stress responses [45], [46], [47], [62], [63], [64], [65]. In fungi, the roles of the transcription factors containing the Myb domain remain largely unknown. However, from the limited studies available, Myb family proteins still show functional diversity and play particularly important roles in cell differentiation and proliferation [49], [50].

In this study, through gene deletion, genetic complementation, and overexpression approaches, we characterized the novel putative transcription factor MYT2, which has functions in various developmental stages including vegetative growth, conidia production, spore morphogenesis, virulence, toxin production, and perithecium development in *G. zeae*. Interestingly, the deletion of *MYT2* resulted in a larger perithecium, while its overexpression resulted in a smaller perithecium when compared to the wild-type strain. Additionally, the ascospores produced by each strain had a relatively consistent perithecial volume. Because MYT2 contains the Myb DNA-binding domain and is localized in nuclei, MYT2 might have an important regulatory role as a transcription factor for the regulation of genes required for cell proliferation and differentiation during perithecium development in *G. zeae*. Moreover, Sordariomycetes-specific conservation of MYT2 demonstrates a conserved function for perithecial development.

MYT2 seems to be a negative regulator for perithecial size differentiation in *G. zeae*. The perithecial size difference shown in the *MYT2* deletion and overexpression mutants suggests that the *MYT2* expression level is negatively related to perithecium size. The *MYT2* transcriptional profile during sexual development in the wild-type strain also supports its function as a negative regulator of perithecium size. The *MYT2* expression level was the highest at 5 d after sexual induction when the perithecial wall had mostly matured and perithecial cell wall proliferation needed to be stopped. Many previously characterized genes related to sexual development are highly expressed from the beginning of sexual induction and increase expression as the perithecia mature, much like *MYT2* [25], [66]. Because the “giant perithecium” in the *MYT2* deletion mutant is a novel mutant phenotype, further characterization of the regulons under MYT2 control may reveal a novel pathway of perithecial development.

Similar to other proteins containing the Myb DNA-binding motif in fungi [48], [49], [50], our results suggest that MYT2 is also related to cell differentiation and proliferation in various developmental stages. The deletion and overexpression of *MYT2* resulted in enhanced and reduced vegetative growth, respectively, which is similar to the results seen for perithecial development. Compared to the wild-type strain, the *MYT2* deletion mutant produced bigger spores, while the overexpression mutant produced smaller spores (Table 2). These results indicated that MYT2 is a suppressor for cell proliferation in various developmental stages in *G. zeae*. Decreased MYT2-GFP expression during conidial germination also supports our hypothesis (Figure 7B). Taken together, MYT2 negatively affects cell proliferation during perithecial development.

There could be two kinds of possibilities to regulate the size of perithecium by MYT2. First, MYT2 could stop cells from dividing at certain point during perithecial development to control the numbers of cells. Second, it could arrest the growth of differentiated cells to regulate the size of individual cell. Our observation showed that the cell size of perithecia formed by myt2 selfing were not different from that of wild-type strain, suggesting that MYT2 is involved in the former case. Several previous works also support that the fungal Myb-domain containing transcription factors regulate cell division. For example, cdc5p of *Schizosaccharomyces pombe* was found to be essential for G_2_/M progression [67], and Reb1 of *S. pombe* is required for fertility. Reb1was originally found to be involved in the termination of ribosomal RNA (rRNA) transcription through binding to 3′ end of the rDNA-coding region [68]. The binding of Reb1 also blocks DNA replication, giving rise to two natural rDNA replication fork barriers (RFBs) [69]. Recently, it was reported that Reb1 binds to a upstream of *ste9* ^+^, resulting in *ste9* ^+^ up-regulation and G1arrest in response to nitrogen starvation [48].

Overexpression of *MYT2* influenced most of the observed phenotypes in the fungus including vegetative growth, sexual development, trichothecene production, and virulence. As a suppressor for cell proliferation, excessive expression of *MYT2* might negatively affect conidia production, although *MYT2* deletion failed to cause a mutant phenotype in conidiation. In trichothecene production, however, we quantified total trichothecenes based on biomass to reduce the effects of decreased mycelial growth on the result. Markedly reduced transcript accumulations of the genes involved in trichothecene production in the *MYT2* overexpression mutant demonstrated that MYT2 additionally functions as a transcriptional repressor for these genes, either directly or indirectly (Figure 6).

The MYT2OE mutant also demonstrated a defect in wheat head virulence. We suspected that a reduction in vegetative growth and trichothecene production, of the MYT2OE mutant would be the reason for reduced virulence [70]. However, the *MYT2* deletion mutant also showed reduced virulence even though radial growth was increased and trichothecene production was similar to the wild-type strain. *G. zeae* virulence is frequently altered by changed hyphal characteristics and the absence of secreted enzymes [23], [32], [60], [71]. Because the mycelial colony of the *MYT2* deletion mutant differed from the wild-type strain, the *MYT2* deletion mutant appears to possess a defect in other biological functions required for virulence.

One of the important steps in the sexual differentiation process is the morphological transition from spherical pre-fruiting bodies (protoperithecia) to flask-like fruiting bodies (perithecia). Much effort has been put forth to understand this developmental stage. Several developmental mutants that arrest after protoperithecia formation were selected and designated as *pro* series in *S. macrospora*. The perithecial morphogenesis of another eight sexual developmental mutants blocked at different stages during perithecia formation has recently been described in detail [9]. However, none of these mutants produce larger perithecia than the *MYT2* deletion mutant. Therefore, further in-depth studies of the regulatory roles of MYT2 in perithecial morphogenesis will provide a novel angle for understanding sexual development in filamentous fungi.

## Supporting Information

Table S1 Primers used in this study.
(PDF)
