# Supplementary material for: A Putative Transcription Factor MYT2 Regulates Perithecium Size in the Ascomycete Gibberella zeae
Source: PLoS One. 2012 May 23;7(5):e37859. doi: 10.1371/journal.pone.0037859 (PMC3359310; doi:10.1371/journal.pone.0037859)
Supplement: Table S1 — Primers used in this study. (PDF) [file pone.0037859.s001.pdf]

Table S1 Primers used in this study

Primer	Sequence(5’→3’)	Description
MYT2-5F	GGGGGTCTCTATATCGCTGTGTTCG	Forward and reverse primers for amplification of 5' flanking region of *MYT2* with tail for geneticin resistance gene cassette fusion
MYT2-5R	<u>GCACAGGTACACTTGTTTAGAG</u>CATGGTCATACGCTGCTGGTTGT	
MYT1-3F	<u>CCTTCAATATCATCTTCTGTCG</u>GTCTAACACCCGGCACTCACTACCT	Forward and reverse primers for amplification of 3' flanking region of *MYT2* with tail for geneticin resistance gene cassette fusion
MYT1-3R	TCGACATTATCTTCCTCCCCTACGG	
MYT1-5N	CCCCCGCGCCTGTTAGTCC	Forward and reverse nest primers for third fusion PCR for amplification of *MYT2* deletion construct
MYT1-3N	TGCCTTGAACATGCTTTACGAACC	
MYT2-seq1	TCCCCTCCAAGACACCTAATGCTT	For RACE-PCR of *MYT2*
MYT2-seq2	GGCTGCATTTGCTGATACGAGTT	
pPRN3-N-For	GTCGAAAATTCAAGACAAGG	For RACE-PCR
pPRN3-N-Rev	AAGCGTGACATAACTAATTAC	For RACE-PCR
MYT2-5R GFP	<u>GAACAGCTCCTCGCCCTTGCTCAC</u>GACACCTCGTCCTGGCCTGTTAAT	Reverse primer for amplification of 5’ flanking region and ORF of *MYT2* with tail for *GFP* tagging complementation
MYT2-5R OE	<u>GATAGTGGAAACCGACGCCCC</u>GAGGCGGATGTGTAGTAGCGAGTAGT	Reverse primer for amplification of 5’flanking region of *MYT2* with tail for *gen* tagging overexpression
MYT2-3F OE	<u>CGGCATGGACGAGCTGTACAAG</u>ATGCCAAAACATACTCGGGGTCCTAG	Forward primer for amplification of *MYT2* ORF with tail for *EF1α promoter* tagging overexpression
MYT2-3R OE	GCTGGGTAATGGCTGGTCTCCTAT	Reverse primer for amplification of *MYT2* ORF for overexpression
MYT2-3N OE	AACCAGAAAACTGTGCGATGATGTGT	Reverse nest primer for third fusion PCR for amplification of *MYT2* overexpression construct
Tri5-realtime-F	GCCATTTTGGACCTTTCTGCTCATT	For realtime-PCR of *Tri5*
Tri5-realtime-R	GCCATAGAGAAGCCCCAACACAAT	
Tri6-realtime-F	GGCAACCATTCAAGCGCTTTTTCT	For realtime-PCR of *Tri6*

Tri6-realtime-R	CACCCTGCTAAAGACCCTCAGACATT	
MYT2-realtime-F	GGCTATGGCGGTCACGGATACT	For realtime-PCR of *MYT2*
MYT2-realtime-R	CCTCGTCCTGGCCTGTTAATGAGAT	
Cyp1-realtime-F	TCAAGCTCAAGCACACCAAGAAGG	For realtime-PCR of *Cyp1*
Cyp1-realtime-R	GGTCCGCCGCTCCAGTCT	
